# Novel and ultra-rare damaging variants in neuropeptide signaling are associated with disordered eating behaviors

**DOI:** 10.1371/journal.pone.0181556

**Published:** 2017-08-28

**Authors:** Michael Lutter, Ethan Bahl, Claire Hannah, Dabney Hofammann, Summer Acevedo, Huxing Cui, Carrie J. McAdams, Jacob J. Michaelson

**Affiliations:** 1 Eating Recovery Center of Dallas, Plano, Texas, United States of America; 2 Department of Psychiatry, University of Iowa, Carver College of Medicine, Iowa City, Iowa, United States of America; 3 Department of Psychiatry, University of Texas Southwestern Medical Center, Dallas, Texas, United States of America; 4 Department of Pharmacology, University of Iowa, Carver College of Medicine, Iowa City, Iowa, United States of America; Universite de Rouen, FRANCE

## Abstract

**Objective:**

Eating disorders develop through a combination of genetic vulnerability and environmental stress, however the genetic basis of this risk is unknown.

**Methods:**

To understand the genetic basis of this risk, we performed whole exome sequencing on 93 unrelated individuals with eating disorders (38 restricted-eating and 55 binge-eating) to identify novel damaging variants. Candidate genes with an excessive burden of predicted damaging variants were then prioritized based upon an unbiased, data-driven bioinformatic analysis. One top candidate pathway was empirically tested for therapeutic potential in a mouse model of binge-like eating.

**Results:**

An excessive burden of novel damaging variants was identified in 186 genes in the restricted-eating group and 245 genes in the binge-eating group. This list is significantly enriched (OR = 4.6, p<0.0001) for genes involved in neuropeptide/neurotrophic pathways implicated in appetite regulation, including neurotensin-, glucagon-like peptide 1- and BDNF-signaling. Administration of the glucagon-like peptide 1 receptor agonist exendin-4 significantly reduced food intake in a mouse model of ‘binge-like’ eating.

**Conclusions:**

These findings implicate ultra-rare and novel damaging variants in neuropeptide/neurotropic factor signaling pathways in the development of eating disorder behaviors and identify glucagon-like peptide 1-receptor agonists as a potential treatment for binge eating.

## Introduction

Eating disorders (EDs), including anorexia nervosa (AN), bulimia nervosa (BN), and binge-eating disorder (BED), form a class of mental illnesses characterized by severe disturbances in body image and meal patterns. While the risk of developing an eating disorder is highly heritable [1], suggesting that genetic variations play an important role in the pathogenesis of EDs, the genetic basis of EDs is incompletely understood. A spectrum of variations exist in the population that account for the genetic risk of developing a complex illness, ranging from common variants that confer low risk to rare variants of very high penetrance [2].

To date, genome-wide association studies and candidate gene approaches designed to identify common variants have yielded mixed results. A genome-wide association study of 5551 individuals with AN and 21,080 control subjects failed to identify any associations with genome-wide significance [3]. Likewise, an association study analyzing 182 candidate genes in 1,085 subjects with AN also did not achieve statistical significance for any individual SNP or haplotype block [4]. A more recent study using targeted sequencing, however, implicated the gene epoxide hydrolase 2 as increasing the risk of developing AN [5].

As a complementary approach designed to identify rare variants that confer high risk, we previously utilized familial segregation and whole exome sequencing in two large families with multiple members affected by EDs [6]. We identified two rare missense mutations, one in the estrogen-related receptor alpha (*ESRRA*) gene and one in the histone deacetylase 4 (*HDAC4*) gene, which increase the risk of developing an ED. In the present study, we performed whole-exome sequencing on 93 unrelated individuals affected by an ED as an alternative approach to identifying additional biological pathways with an excessive burden of rare, damaging mutations. As there is meaningful clinical overlap in the presentation of certain eating disorders such AN and BN, and BN and BED, and because a significant sub-set of patients will convert diagnosis in their lifetime [7–9], we elected to focus our analysis on eating-disorder related behaviors instead of diagnosis. We therefore chose to analyze the 38 patients with restricted-eating together (anorexia nervosa- restricting) and the 55 patients with binge-eating episodes together (anorexia nervosa- binge/purge, bulimia nervosa, binge eating disorder) to improve the behavioral homogeneity of the groups. We find that distinct neuropeptide/neurotrophic factor signaling pathways have an excessive burden of novel damaging mutations that may contribute to the risk of developing disordered eating behaviors.

## Methods

### Patient recruitment

The Institutional Review Board of the University of Iowa approved all procedures (#210211783). Participants in the study were recruited from Inpatient and Partial Hospitalization programs at an academic medical center, as well as from advertising within the University community from June 2013-June 2015. The age range for patients in the study was 18–60 with a median age of 24. No participants withdrew from the study. Following initial screening, all participants provided informed written consent and received a structured clinical interview for DSM-IV Axis I disorders (http://www.scid4.org/), and were assigned a diagnosis of anorexia nervosa- restricting, anorexia nervosa- binge/purge, bulimia nervosa, or binge-eating disorder (S1 Table). Patients with a diagnosis of anorexia nervosa- binge/purge must have met criteria for binge episodes (not solely purging behaviors) to be included in the analysis for the binge-eating group. Previous ED diagnoses were not determined, so we cannot exclude the possibility of lifetime diagnostic conversion between the restricted-eating and binge-eating groups. Genomic DNA was collected and stored. The first 98 individuals who qualified for the study received whole exome sequencing performed at the Broad Institute (Cambridge, MA). Three individuals were excluded from analysis after quality control measures indicated the samples were cross-contaminated.

### Whole exome sequencing

Exome capture was performed at the Broad Institute using the Broad Standard Exome platform. Paired-end, 76bp libraries were prepared for each sample and these were sequenced to a mean depth of 95X coverage over the captured regions (29 Mb baited, > 91% of captured regions had coverage > 20X). Sequencing was performed on either an Illumina HiSeq 2000 v3 or HiSeq 2500. Sequencing reads were aligned to the hg19 reference genome using BWA [10] according to the Broad Standard Exome pipeline.

### Variant calling and filtering

Variants were called and genotyped in the entire cohort using Platypus [11]. All variant sites were then annotated with ANNOVAR [12]. Variants were excluded from further analysis if they fell outside exonic, UTR, or splicing regions, or if Platypus marked them with the QD, Q20, badReads, or alleleBias FILTER flags.

These variants were further filtered out if they had been observed in the 1000 Genomes Project [13], dbSNP (build 138), or Exome Variant Server [14]. Additionally, to enrich for potentially damaging variation, variants with a phred-scale CADD [15] score of less than 15 were excluded from further analysis (15 is the threshold suggested by the creators of CADD). The remaining variants in all subsequent analyses are therefore novel and expected to have functional consequences.

### Novel variant significance testing

To determine whether any gene showed enriched burden of potentially damaging variants, we calculated the number of novel, damaging variants (using the criteria above) appearing in the ExAC database [16] (data contributed by psychiatric exomes were excluded). Using these ExAC counts (genewise deleterious variants and the corresponding number of individuals contributing to that count) as a comparison group, we were able to test each gene hit in our cohort for excessive burden using the prop.test() function in R [17] (this performed a one-sided test for equal proportions in count data). P values were corrected for multiple testing using the Benjamini-Hochberg procedure [18].

### GO and expression evidence

To prioritize the list of genes identified in the exome analysis, we used machine learning to develop evidence scores based on the similarity of all genes to genes previously identified in connection with eating disorders or appetite. Two types of evidence were considered: expression (using the BrainSpan[19] gene expression data) and Gene Ontology[20] (GO) functional annotations. Each of these data sets were used as predictors in a machine learning task to discriminate candidate ED genes (genes supported by citations in three or more papers matching “eating disorder” or “appetite” searches via PubMed; S2 Table) from a set of 1000 of the most highly cited genes that had never been cited in connection with either “eating disorder” or “appetite”. The randomForest R package[21] was used to accomplish this task (ntree = 100, default settings for all other parameters), one classifier for each evidence form (expression and GO). Vote proportions for the “ED candidate” class were used as evidence scores (0 being lowest and 1 being highest). All non-training genes were scored by each classifier, and scores for the training set were taken from the out-of-bag estimates from the respective classifier.

### Network analysis

**Fig 1:**
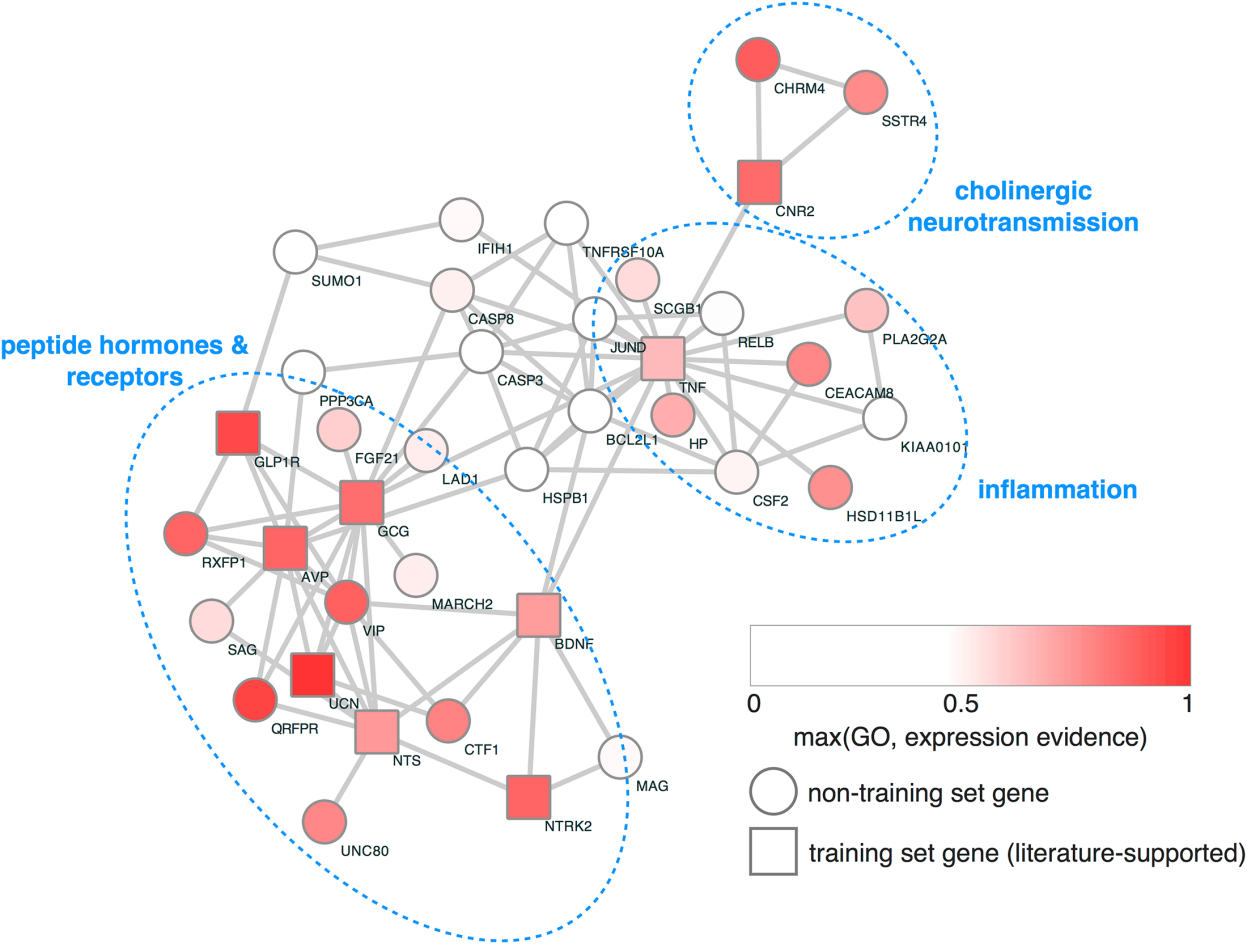
Network of genetically-associated genes having additional evidence for involvement in appetite or feeding behaviors. Genes showing significantly increased burden (FDR < 0.1 for either binge or restricting phenotypes) were projected onto the STRING functional network, and nodes were shaded according to their expression/GO evidence supporting their role as a putative ED gene (see Methods for details on Random Forest classifier). Square-shaped nodes were shown to have supporting evidence in an automated analysis of the literature (i.e., they are training set genes for the Random Forest classifier).

We projected all genes with an FDR < 0.1 for either test (binge or restricted) onto the STRING v10 network [22]. We then extracted the connected component of all training set positive examples of ED/appetite genes (see above) and their neighbors. Nodes (genes) were colored according to their RF classifier evidence (maximum of expression and GO evidence, above) for being an ED gene, and this subnetwork was interpreted visually (see Fig 1).

### Sanger sequencing

Sanger sequencing was performed to confirm several key variants using the following primer pairs: Neurotensin: Exon2 (CTCCCAGTGATCAATAGCTGTC GGAAATGCCTCTCAAGGAAC), Exon3 (ccctaaatgtgatggtttagcac TGTTGACTTCAAAACATGGC), Exon4 (CGTATCTCACCTACATATGTCTTGC GTGTGTTTGTCACATTTTCACAC); UNC80: Exon11 (CTAACAGCCCCACACTCTCC GAAAACACAGGAAGCGGATTAC), Exon14 (CAAAAGGACCTCAGTGACAAAG CATTCTTAAACAGAAGGGAAGG), Exon18 (CAACAGAGTGTTTTGTTTTATGAAG TTCTTCCAGAGTGAAGTGCATAG), Exon23 (ACCACCCTGGGAAATATGG cccaaagtgtttccttcagtacc), Exon25 (TGTCCATCAAACTATGCATTTC GAAACCCAGACACACGCC), Exon30 (GGGACGTGGCTTTTAAGTTTC CTTGGATCAGTAACAGCAGTGG), Exon45 (AATCATACCACAGCATTGCG TCATGGCAGAGAATTCCTTACTC); Glucagon: Exon3 (TTGAAATACTCTAGATGCCTGCC GGGCTTATGGGCACTATTTG); GLP-1R: Exon4 (ATAGCCCTCAGAATGGGGAG CAAGGGTCTTTGCTCCAGTG), Exon10 (TCTGTGCCTGCACCTGAG TAGAAGGAAAGGGGCACTTG).

### Animal usage

All animal procedures were performed in accordance with University of Iowa Institutional Animal Care and Use Committee guidelines. Mice were handled in accordance with the Guide for the Care and Use of Laboratory Animal, as adopted by the U.S. National Institutes of Health. Specific protocols were approved by the Institutional Animal Care and Use Committee. Female C57BL6 mice were housed in the University of Iowa vivarium in a temperature-controlled environment (lights on: 06:00–18:00) with ad lib access to water and regular chow (7913 NIH-31 modified open formula mouse sterilized diet, Harlan-Teklad, Madison, WI) or exposed to high fat diet (42.8% calories from fat, TD.88137, Harlan-Teklad) as noted. Binge-like eating episodes were achieved in mice using a protocol described by [23]. Briefly, mice have weekly access to HFD for 24 hours (starting at ZT 0700) along with continuous access to regular chow. Mice develop stable ‘binge-like’ episodes in which they consume between one-third to one-half of their daily calories during the first two hours of access. After stable binge-like feeding was achieved, mice received daily handling and saline injection I.P. for five days. On the test day, exendin-4 (#1933, Tocris) was dissolved in sterile saline and injected at 2.4 micrograms/kg body weight (in a volume of 100 microliters/ 20 grams of body weight) at ZT 0630. This dose was selected because it affects consumptive behavior without affecting locomotor activity [24,25]. HFD intake was measured from ZT 0700–0900. At the conclusion of the study, mice were sacrificed by CO2 asphyxiation.

## Results

### Prioritization of novel damaging variants

Whole exome sequencing was performed for 38 individuals with anorexia nervosa-restricting (‘restricted-eating’ group) and 55 individuals with anorexia nervosa- binge/purge, bulimia nervosa, and binge eating disorder (‘binge-eating’ group). We generated a list of called variants predicted to be damaging by CADD score, which were then compared to the Exome Aggregation Consortium (ExAC) database to identify novel, putatively functional (presumed damaging or hypoactive) variants. Hereafter we refer to these as “damaging variants”. The burden of damaging variants was then calculated for each gene and this was compared to the expected value (i.e., the observed count in ExAC) using prop.test() in R.

We next conducted an unbiased, data-driven analysis to prioritize damaging variants most likely to contribute to the risk of developing an ED. First, we identified all genes mentioned in three or more papers in the PubMed database that mention either "appetite" or "eating disorder" (S2 Table). Nine genes from this training set were found to have an increased burden of novel damaging mutations in our ‘restricted-eating’ and ‘binge-eating’ groups representing a highly significant, more than four-fold enrichment for known ED genes (OR = 4.2, P< 0.001).

Next we sought to prioritize the associated genes by developing an integrated measure of external evidence supporting the gene as being involved in EDs. First, we assembled a training set of genes comprised of our previously described literature-based list of ED genes (positive class) and 1000 of the most highly cited genes, which had no citations in connection with either appetite or eating disorders (negative class). We trained a classifier to discriminate these two classes using the BrainSpan gene expression data. Predictions were then made on the remaining genes on the list in order to calculate a score for "expression evidence" in which higher values mean that the gene has a similar development and neuroanatomical expression pattern to ED genes. This process was then repeated using GO annotations as predictors instead of gene expression data, to calculate a score for "GO evidence" such that high-scoring genes share similarity with ED genes in terms of their functional annotations in GO. The expression evidence score and the GO evidence score were then used to prioritize the binge-eating and restricted-eating gene lists for potential involvement in the development of disordered eating behaviors.

### Network analysis

Unbiased network analysis of the genes identified on the restricted-eating and binge-eating groups (see Methods) found that three distinct themes emerge: 1) neuropeptide hormones and their receptors, 2) inflammation, and 3) cholinergic neurotransmission. Neuropeptide signaling has strong face validity in that many neuropeptide hormones affect appetitive behaviors and body weight homeostasis. Additionally, multiple ligand-receptor pairs were identified by our analysis (including BDNF-TRKB, GLP1-GLP1R, and NTS-UNC80, reducing the probability that these genes were found by chance alone. Finally, inflammation and cholinergic signaling have external supporting evidence associating their function with appetite suppression and motivated behaviors, respectively.

### Novel variants in restricted-eating group

In total 186 genes were identified with damaging variants occurring at a significantly higher rate than predicted, including six genes from the ‘ED training’ list of genes previously implicated in appetite and EDs (S3 Table). Notably, several neuropeptides ranked very high in their GO and expression evidence scores, including vasoactive intestinal peptide (*VIP*), urocortin (*UCN*), and neurotensin (*NTS*) (S3 Table). In total 5 genes from the restricted-eating group are neuropeptide transmitters or receptors, including the neuropeptide transmitter neurotensin, the gene with the highest number of novel damaging mutations (4 observed vs. 0.1 predicted) in the restricted feeding group.

**Fig 2:**
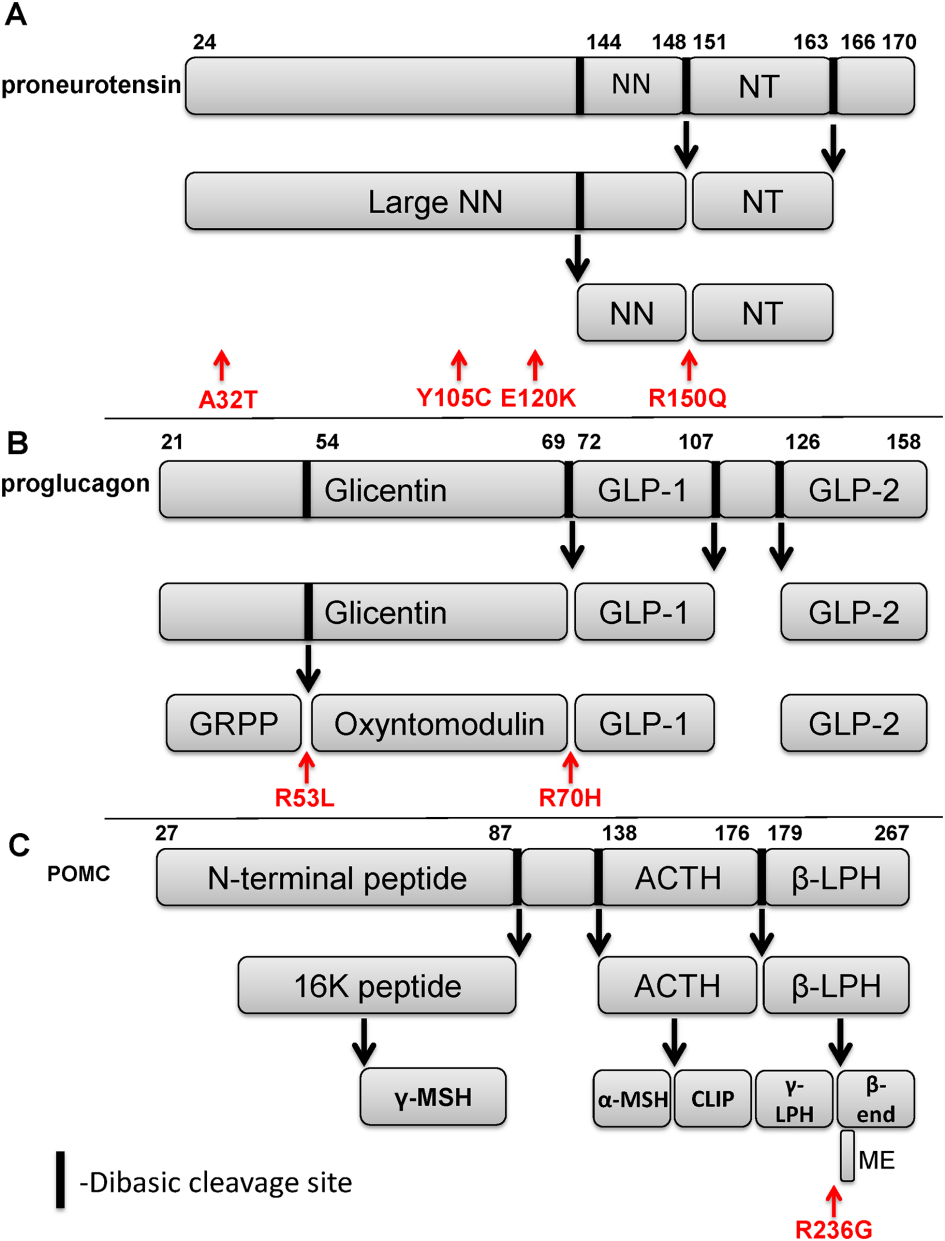
Schematic representation of select peptide neurotransmitters. (A) proneurotensin, (B) proglucagon, and (C) proopiomelanocortin. Proteolytic cleavage sites highlighted with arrows. Variants observed in our sample are labeled in red. NN- neuromedin N, NT- neurotensin, GLP-1- glucagon-like peptide 1, GLP-2- glucagon-like peptide 2, GRPP- glicentin-related polypeptide, ACTH- adrenocorticotrophic hormone, β-LPH- beta lipotrophin, γ-MSH- gamma melanocyte stimulating hormone, α-MSH- alpha melanocyte stimulating hormone, CLIP- corticotrophin-like intermediate peptide, γ-LPH- gamma lipotrophin, β-end- beta endorphin, ME- met-enkephalin.

Because biologically active neuropeptides are derived from proteolytic cleavage of a propeptide, we analyzed the observed variants to better understand the functional consequences. One variant in the neurotensin gene results in an arginine to glutamine substitution at position 150 that ablates the dibasic KR site required for cleavage by prohormone convertases and generation of the neuropeptide neurotensin (Fig 2A). The three other variants we observed occur in the portion of the propeptide that is processed into the large neuromedin N neuropeptide, which also signals through the neurotensin receptor [26]. Because we identified four variants predicted to affect ligands for the neurotensin receptor, we also searched for novel damaging variants in the neurotensin receptor 1 gene in the cohort and found one individual who harbored a frameshift deletion that introduced a stop codon near the start of exon 2 (Table 1). Of note, this individual also has a strong family history of AN with a daughter and grand daughter with AN who also share the variant. In total, 5/38 individuals in our restricted eating group had damaging variants in neurotensin or neurotensin receptor 1.

**Table 1:** Variants in neuropeptide processing pathways identified in our patient samples. Chr- chromosome, Ref- reference allele, Alt- alternate variant, CADD- Combined Annotation Dependent Depletion phred-like score.

Restricted-eating		Start	End	Ref	Alt	CADD	ExAC MAF	RS#
	Chr							
**NTS**	12	86270438	86270438	G	A	15.89	NA	NA
	12	86272301	86272301	A	G	16.85	NA	NA
	12	86272345	86272345	G	A	23.3	NA	NA
	12	86276089	86276089	G	A	29.9	NA	NA
**NTSR1**	20	61386033	61386038	GCAGGT	G	NA	NA	NA
**UCN**	10	5416075	5416075	T	C	21.3	5.03E-05	rs142836326
**VIP**	6	153075342	153075342	A	G	22.5	NA	NA
**VIP**	6	153077340	153077340	G	A	19.39	NA	NA
**Binge-eating**								
**GCG**	2	163003908	163003908	C	T	18.38	NA	NA
	2	163003961	163003962	GC	AA	NA	NA	NA
**GLP1R**	6	39033595	39033602	GAGGGGAA	AAGGGGAG	NA	NA	NA
	6	39046934	39046934	A	G	28.7	NA	NA
**BDNF**	11	27680107	27680107	G	T	20.557	NA	NA
**NTRK2**	9	87325623	87325623	C	A	18.764	NA	NA
	9	87549182	87549183	AG	CC	NA	NA	NA
**QRFPR**	4	122301595	122301597	CCA	ACC	NA	NA	NA
**UNC80**	2	210682673	210682673	G	A	27.5	NA	NA
	2	210690770	210690770	G	A	15.64	NA	NA
	2	210699658	210699658	G	A	24.6	NA	NA
	2	210737632	210737632	G	A	23.6	NA	NA
	2	210745728	210745728	G	C	18.51	NA	NA
	2	210778652	210778652	G	A	32	NA	NA
	2	210809854	210809854	C	A	27.3	NA	NA
**AVP**	20	3063808	3063808	G	A	21.8	NA	NA
**Additional**								
**POMC**	2	25384048	25384048	G	C	21.6	0.00231	rs28932472

### Novel variants in binge-eating group

245 genes were identified with damaging variants occurring at a significantly higher rate than predicted in the binge-eating group including six genes from the ‘ED training’ list of genes previously implicated in appetite and EDs (S4 Table). Once again neuropeptides and neuropeptide receptors were ranked highly by the expression and GO evidence scores, including, glucagon (*GCG*), glucagon-like peptide 1 receptor (*GLP1R*), arginine vasopressin (*AVP*) and pyroglutamylated RF amide peptide receptor (*QRFPR*) (Table 1). The variant we observe in QRFPR is of unclear significance, as we called it as a trinucleotide indel, but it is represented in ExAC as two independent SNPs (rs55693553, rs34270076) that appear to always occur on the same haplotype. These SNPs together would result in a two-amino acid substitution (VV69/70LG). The MAF for both SNPs is 0.012, which would ostensibly be the MAF for the indel we called. Assuming this is the case, our observation of the variant in three out of 93 individuals represents a modest increase over the expectation of one individual in a sample of our size. Additionally, the protein product of *UNC80*, the gene with the highest number of damaging variants in the binge-eating group (6 variants observed vs. 1.6 predicted), is a component of the sodium leak channel, non-selective (NALCN), an ion channel that is regulated by the activity of the peptide neurotransmitters substance P and neurotensin [27,28].

Like neurotensin, preproglucagon, the protein product of the glucagon gene, is proteolytically cleaved to generate several neuropeptides in brain including oxyntomodulin, glicentin, glucagon-like peptide 1 (GLP-1) and glucagon-like peptide 2 [29]. Analysis of the two variants found in the glucagon gene show that the first variant ablates an arginine within the dibasic cleavage site required for proteolytic processing and generation the oxyntomodulin peptide (Fig 2B), an agonist for the GLP-1 receptor (GLP-1R) in the brain and gut, while the second damaging variant also is a missense variant that replaces an arginine within the cleavage site required for generation of GLP-1 [29]. Because both oxyntomodulin and GLP-1 are agonists for the GLP-1 receptor, 4/55 individuals in the binge-eating group harbor mutations that affect GLP-1 receptor signaling.

We observed damaging variants in both the neurotrophic small protein brain-derived neurotrophic factor (*BDNF*) and one of its receptors, the neurotrophic tyrosine kinase receptor, type 2 gene (also known as TrkB protein), both within the network region defined by peptide hormones and receptors (Fig 1). Of note, *BDNF* has previously been implicated in food intake and body weight regulation [30,31], as well as the risk of developing an ED [32].

Because neuropeptide transmitters and receptors were over represented on both lists, we assessed several other neuropeptides with a known role in appetite regulation including the proopiomelanocortin (*POMC*) gene. Three individuals in the cohort were found to have the rs28932472 SNP (c706g) in *POMC* (Table 1). This SNP has previously been found in one child from a French cohort of children with severe obesity, although disordered eating behaviors were not assessed [33]. While this variant did not appear on the list of novel variants because it has been reported previously, its low frequency (minor allele frequency 0.00231 in ExAC vs. 0.0154 in this sample) may suggest negative selection. Analysis of the variant reveals an arginine to glycine substitution at position 236 of the propeptide (Fig 2C), which ablates a dibasic cleavage site required for processing of the endogenous opioid peptides beta-endorphin and met-enkephalin, two peptide neurotransmitters that have previously been implicated in hedonic feeding [34,35].

### GLP-1 receptor agonists in a mouse model of binge-like eating

**Fig 3:**
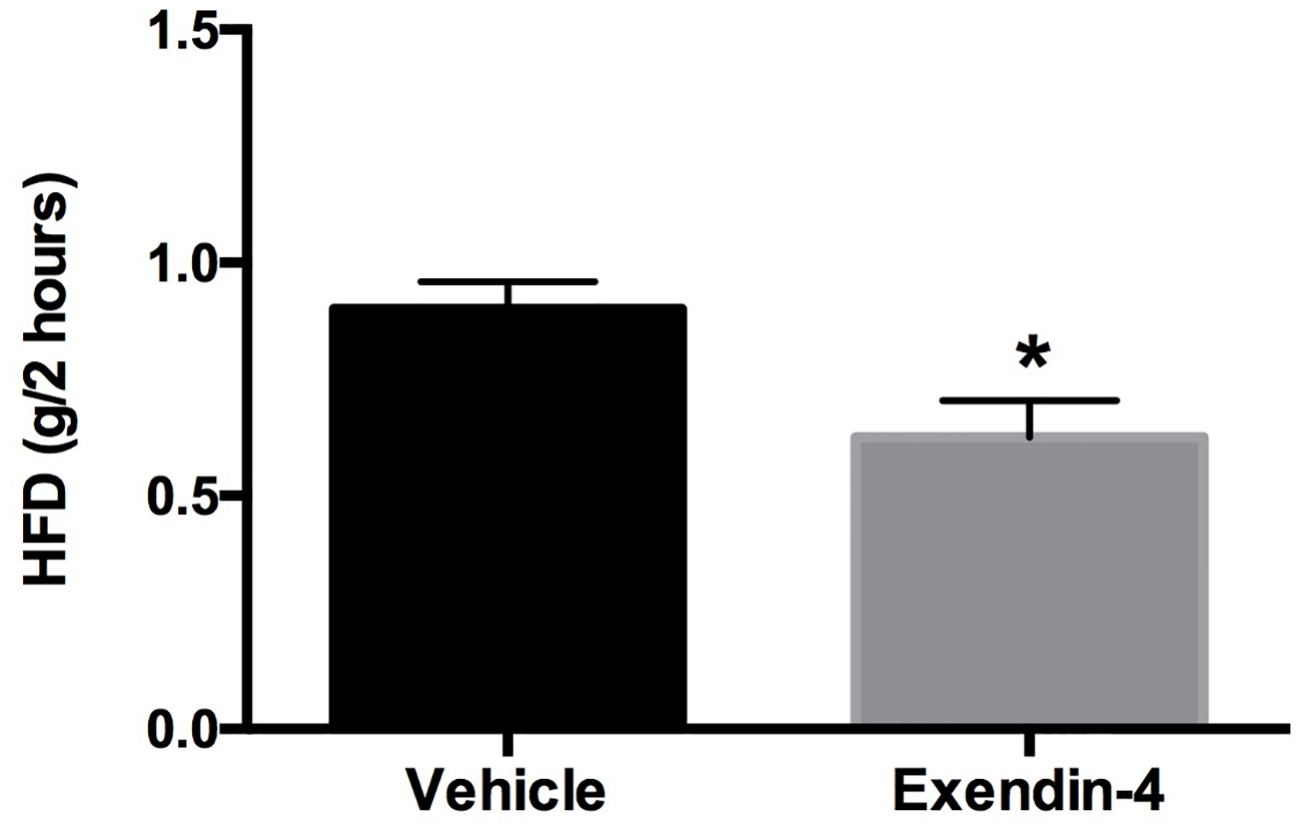
Exendin-4 administration in a model of ‘binge-like’ eating. Twelve-week-old wild-type female mice were placed on a protocol with intermittent access to HFD. After stable episodes of ‘binge-like’ feeding were achieved, mice received acute administration of exendin-4 (2.4 micrograms/kg) or vehicle 30 minutes prior to access to HFD. Two-hour HFD intake is significantly reduced after exendin-4 administration (n = 8, p = 0.0497 by Student’s t-test). Data presented as mean ± SEM with *p < 0.05 considered significant.

Finally, GLP1 signaling regulates food intake [36] and GLP1 levels have been reported to be low in patients with BN [37,38]. Therefore we tested if pharmacologic activation of GLP1 receptors by exendin-4 decreases food intake in a model of binge eating-like behaviors in mice. Peripheral administration of a low physiological dose of exendin-4 resulted in a significant decrease in the size of the ‘binge-like’ episode in female mice consistent with a potential therapeutic role in the treatment of binge-like behaviors (Fig 3).

## Discussion

In the current study we report for the first time the results of whole exome sequencing of 93 individuals with eating disorders to biological pathways with an excess load of novel and ultra-rare damaging variants. Network analysis of genes with significantly elevated burden (Fig 1) revealed dense connectivity among neuropeptide transmitters and their receptors, implicating them in the genetic etiology of EDs. Of particular note, mutations that ablate an arginine residue within the dibasic cleavage site recognized by prohormone convertases are surprisingly common in individuals with EDs, with four separate mutations observed in three genes.

Pre-clinical studies support a potential role for these neuropeptide-signaling pathways in the development of eating disorder related behaviors. In particular, multiple studies have identified an important role for both neurotensin-neurotensin receptor signaling and GLP1-GLP1R signaling in regulation of the brain regions implicated in motivated feeding behaviors. For example, neurotensin is highly expressed in the central nucleus of the amygdala (CEA), the lateral hypothalamic area (LHA), and the paraventricular nucleus of the hypothalamus (PVN) [36,39]. These regions are highly interconnected, including neurotensin-expressing neurons in the CEA that project strongly to the LHA [39], suggesting that CEA-LHA-PVN may form a neural network that regulates motivated feeding behaviors. In support of this model, neurotensin signaling is required for weight gain induced by consumption of high-fat food[40] and deletion of leptin receptors from neurotensin-expressing neurons in the LHA increases food intake and decreases locomotor activity[41], suggesting that neurotensin is important for mediating behavioral adaptations to starvation. Mice lacking neurotensin receptor 1 also exhibit extreme phenotypes of decreased intake of regular chow, but increased consumption of highly palatable foods [42], reminiscent of patients with AN-binge/purge subtype who restrict intake of regular meals and binge on calorically dense foods.

Several studies also support a role for GLP1-GLP1R signaling in modulating activity of this network. A GLP1R antagonist blunts the anorectic effect of leptin [43], suggesting that GLP1R signaling is important for coordinating behavioral responses to changes in energy homeostasis. GLP1R is also expressed in the CEA, LHA, and PVN, and administration of the GLP1R agonist exendin-4 induces neuronal cFos levels (a marker of neuronal activation) and neurotensin mRNA expression in PVN neurons [36,44].

Clinical and translational studies have also reported a link between GLP-1 signaling and binge-eating behaviors. For instance, GLP-1 decreases food intake induced by deletion of estrogen receptor alpha in serotonergic neurons of female mice [45]. Furthermore, GLP-1 secretion after meals is blunted in patients with BN [37,38], and administration of the GLP-1 agonist liraglutide reduced binge-eating episodes in a pilot study of non-diabetic obese patients with subclinical binge eating [46]. As all four participants in this study with variants in *GLP-1* signaling carried a diagnosis of BN, GLP-1 receptor agonists, currently approved for the treatment of diabetes and chronic weight management, may serve to treat a sub-set of patients with BN.

While this study focused on variants within neuropeptide signaling pathways, we also observed an increase in the burden of damaging variants in several other biological pathways potentially relevant to the development of EDs. The second pathway to emerge from our analysis is inflammation (Fig 1). In addition to tumor necrosis factor (*TNF*) itself, several other genes related to TNF activity were found to have an excessive burden of damaging variants including *TNFRSF10A*, *BCL2L1*, *CASP3*, *CASP8*, *CSF2*, and *CEACAM8*. Unlike neuropeptide signaling, most of the genes in the TNF pathway identified by our analysis were clustered in the restricted-eating group (10/16). Dysregulation of TNF signaling has been associated with anorexia and cachexia in a number of medical illnesses including rheumatoid arthritis [47], inflammatory bowel disease [48], and malignancies [49]. A meta-analysis of 22 studies concluded that levels of the pro-inflammatory cytokines tumor necrosis factor alpha, interleukin-6 and interleukin-1b were elevated in patients with AN and were not corrected by weight restoration [50]. Our genetic findings suggest that elevations of these cytokines might be in part due to genetic predisposition to pro-inflammatory states, which may contribute to the development of AN instead of strictly being a consequence of starvation. Case-reports have suggested a potential benefit of TNF-lowering agents in the treatment of patients with AN [51,52] supporting the need for further studies on the subject.

Finally, a small cluster of genes implicated in cholinergic signaling (*CNR2*, *CHRM4*, *SSTR4*) were also identified by our analysis (Fig 1). Of note, a variant in the *CNR2* gene, which encodes the cannabinoid receptor 2, has previously been linked to the risk of developing an eating disorder in a sample of Japanese patients with eating disorders [53], providing further evidence supporting a role for *CNR2* variants in the risk of developing an ED. These genes have been linked to the function of the mesolimbic dopamine signaling [54–58], a pathway in the brain critical to regulation of goal-directed behaviors, such as food intake, which has previously been implicated in the development of EDs [59,60].

Several aspects of the present study that may affect interpretation of the data merit discussion. First, we sequenced only cases and not controls. However, the massive compendia of exome data in ExAC, which includes data from over 60,000 exomes, provides a more complete baseline reference of rare exonic variation than sequencing a comparatively small number of controls. Focusing our analysis exclusively on novel and ultra-rare variants in the ExAC database also eliminated the need to control for differences in ethnicity, as the MAF is essentially 0 for all ethnicities within the control subjects. Nevertheless, ongoing studies examining damaging mutations already reported in the ExAC database will need to take this into account.

While we did not experimentally demonstrate functional consequences of the mutations, the substrate specificity of prohormone convertases has been extensively studied and it has been known for 25+ years that a pair of dibasic residues is required for their cleavage activity. Therefore, we elected not to demonstrate this functional consequence as it has been so well established from the literature.

Finally, while we attempted to select a set of patients that represented clinical populations, it is likely that the group is overrepresented for severe cases. This is partly due to recruitment from Inpatient Hospital Programs, which require greater disease severity for admission. Therefore, replication of our findings in a larger community-based cohort will be necessary to determine how frequently novel or rare variants in peptide neurotransmitters contribute to the risk of developing disordered eating behaviors.

## Supporting information

S1 Table Participant characteristics. (XLSX)

S2 Table Genes cited 3+ times in connection with eating disorders or appetite. (XLSX)

S3 Table Genes from restricted-eating group with increased burden of damaging variants. (XLSX)

S4 Table Genes from binge-eating group with increased burden of damaging variants. (XLSX)

## Abbreviations

AN: Anorexia Nervosa
BED: Binge Eating Disorder
BN: Bulimia Nervosa
CEA: central nucleus of the amygdala
ED: eating disorder
ExAC: Exome Aggregation Consortium
LHA: lateral hypothalamic area
PVN: paraventricular nucleus
